# Increased Baseline Proinflammatory Cytokine Production in Chronic Hepatitis C Patients with Rapid Virological Response to Peginterferon Plus Ribavirin

**DOI:** 10.1371/journal.pone.0067770

**Published:** 2013-07-09

**Authors:** Gabriella Par, Laszlo Szereday, Timea Berki, Laszlo Palinkas, Melinda Halasz, Attila Miseta, Geza Hegedus, Julia Szekeres-Bartho, Aron Vincze, Bela Hunyady, Alajos Par

**Affiliations:** 1 Clinical Centre, First Department of Medicine, University of Pécs, Pécs, Hungary; 2 Clinical Centre, Department of Medical Microbiology and Immunology, University of Pécs, Pécs, Hungary; 3 Clinical Centre, Department of Immunology and Biotechnology, University of Pécs, Pécs, Hungary; 4 Clinical Centre, Department of Laboratory Medicine, University of Pécs, University of Pécs, Medical School, Pécs, Hungary; 5 Department of Pathology, Baranya County Hospital, Pécs, Hungary; 6 Janos Szentagothai Research Centre, Pécs, Hungary; University of Washington, United States of America

## Abstract

**Background:**

Chronic hepatitis C (CHC) patients achieving rapid virological response (RVR) on PEG-IFN/ribavirin (P/R) therapy have high chance of sustained virological response (SVR). To analyze host immunological factors associated with RVR, viral kinetics, phenotype distribution and Th1/Th2 cytokine production by peripheral blood mononuclear cells (PBMC) were studied prior to and during P/R therapy.

**Methods:**

TNF-α, IFN-γ, IL-2, IL-6, IL-4 and IL-10 production by PBMC were measured after Toll-like receptor 4 (TLR-4) or phorbol myristate acetate/Ionomycin stimulation in 20 healthy controls and in 50 CHC patients before receiving and during P/R therapy. RVR was achieved by 14, complete early virological response (cEVR) by 19 patients and 17 patients were null-responders (NR).

**Results:**

Patients with RVR showed an increased baseline TNF-α and IL-6 production by TLR-4 activated monocytes and increased IFN-γ, decreased IL-4 and IL-10 production by lymphocytes compared to non-RVR patients. SVR was also associated with increased baseline TNF-α production and decreased IL-10 levels compared to patients who did not achieve SVR. Baseline IL-2 production was higher in cEVR compared to NR patients. Antiviral treatment increased TNF-α, IL-6 production by monocytes and IFN-γ secretion by lymphocytes and decreased IL-4 and IL-10 production by lymphocytes in cEVR compared to NR patients.

**Conclusion:**

RVR was associated with increased baseline proinflammatory cytokine production by TLR-4 stimulated monocytes and by activated lymphocytes. In null-responders and in patients who did not achieve SVR both TLR-4 sensing function and proinflammatory cytokine production were impaired, suggesting that modulation of TLR activity and controlled induction of inflammatory cytokine production may provide further therapeutic strategy for CHC patients non-responding to P/R treatment.

## Introduction

Hepatitis C virus (HCV) evades the host’s immune response and resists the antiviral action of pegylated interferon-alfa and ribavirin in approximately half of the HCV genotype 1 infected individuals. Beside viral and environmental factors, host factors such as innate and adaptive immune responses are likely to be key players in determining the type of virological response to P/R treatment [1]–[7]. Beside IL28B gene polymorphisms, negative plasma HCV-RNA after 4 weeks of P/R treatment (rapid virological response) is known as the strongest predictor of sustained virological response. [8]–[11]. The immunological mechanisms responsible for rapid virological response (RVR) have not been clarified completely. Liver gene expression profiles of responders differ significantly from those of null-responders (NR), most notable changes were observed in the IFN-stimulated genes (ISG) and in cytokine genes. Although the basal level of hepatic ISG gene expression (e.g. MxA, ISG15) is higher in non-responders than in the sustained virological responder group, in patients with RVR pegylated interferon-alfa treatment induces a strong up-regulation of IFN-stimulated genes [12]. Activation of the endogenous IFN system in CHC is not only ineffective in clearing the infection but also may impede the response to therapy, most likely by inducing a refractory state of the IFN signaling pathway.

Cytokines play an important role in the defense against viral infections determining the pattern of host immune response and inhibiting viral replication [13]. Both pegylated interferons and ribavirin have not only antiviral but also immunomodulatory properties such as alteration of immune functions and Th1/Th2 cytokine balance [14]–[17]. Increased Th2 and altered Th1 cytokine production have been associated with viral persistence and failure of antiviral treatment in CHC [18], [19]. High baseline levels of IL-10 and low levels of IL-12p40 were significantly associated with a non-virological response (NVR) while low pretreatment IFN-γ inducible protein 10 (IP-10) plasma level is also known to predict SVR to therapy [20].

Our hypothesis was that immune cells of patients with RVR may have higher capacity to produce Th1 type cytokines, determining early cytotoxic antiviral innate response compared to patients without rapid virological response. Since Toll-like receptors (TLR) are important in innate immune recognition of viral infection and induction of inflammatory cytokines [21], [22], in this prospective study TLR-4 and phorbol myristate acetate (PMA)/Ionomycin induced Th1/Th2 cytokine production by peripheral blood mononuclear cells (PBMC) was investigated in rapid virological responders, complete early responders and null-responders prior to and throughout 24 weeks of P/R treatment. Baseline cytokine production in patients with sustained virologic response (SVR) and in patients who did not achieve SVR was also analyzed.

## Materials and Methods

### 1. Patients

Fifty treatment-naive patients with chronic HCV hepatitis (25 males, 25 females, mean age 49,8 years) being on 1,5 µg/kg/week of PEG-IFN- α 2b (PegIntron, Schering-Plough) or 180 µg/week of PEG-IFN- α 2a (Pegasys, Roche) plus 1000 or 1200 mg/day (for bodyweight <75 kg or >75 kg, respectively) of ribavirin (RBV) (Rebetol, Schering-Plough or Copegus, Roche) therapy have been studied prior to and after 1, 3 and 6 months of antiviral treatment. All patients had HCV 1b genotype infection. The criteria for CHC were: detectable serum HCV RNA, liver histological examination showing characteristic lesions of CHC. All the patients were HBV and HIV negative and showed no signs of any other chronic liver diseases.

Liver biopsy specimens were obtained before the initiation of the treatment. Knodell and Metavir scores were used to determine the histological activity index and fibrosis.

RVR (undetectable, <50 IU/ml HCV RNA at week 4) was achieved by 28% (14/50), complete early virological response (cEVR, undetectable HCV RNA at week 12) by additional 38% (19/50) of patients and 34% of patients were null-responders. Sustained virological response (SVR) was defined as undetectable serum HCV RNA at 6 months after the end of therapy. 85,7% (12/14) of rapid virological responders, 57,8% (11/19) of complete early virological responders showed SVR. Altogether 46% (23/50) of the patients achieved SVR, 54% (27/50) were non-responders or relapsers considered as non-SVR patients (Table 1.). 20 healthy blood donors formed the control group.

**Table 1:** Patients’ baseline characteristics.

	RVR	cEVR	NR
No. of patients	14	19	17
Age (years) (mean)	49,6±4,7	45,2±1,4	54,7±7,3
Gender(Male/Female distribution)	6/8	12/7	4/13
SVR	12/14	11/19	0/27
BMI	27,4±3,2	28,3±4,5	24,55±4,2
HAI	7,46±2,2	8,26±1,3	7,3±2,2
Fibrosis	2,15±1,3	2,12±1,3	2,9±1,24
ALT	124±61	115±46	125±58
Genotype 1b	14/14	19/19	17/17
HCV RNA (kIU/ml)	393±379	662±358*	942±865**

Baseline HCV RNA levels were significantly lower in rapid virological responders (RVR) compared to complete early virological responders (cEVR) and null-responders (NR). Pretreatment histology and ALT did not differ significantly between study groups. Results are expressed as mean±SE (*p<0,05; **p<0,01).

(SVR = sustained virological response, BMI = body mass index, HAI = Knodell histological activity index, ALT = alanine amino transferase).

Written informed consent was obtained from all patients. The study protocol conforms to ethical guidelines of 1975 Declaration of Helsinki. Approval from the Regional Ethics Committee at the Medical School, University of Pécs, was obtained.

### 2. HCV-RNA Detection and HCV Genotyping

Serum HCV RNA detection and quantification were performed with Roche Cobas Amplicor HCV 2,0 assay (lower limit of detection <50 IU/ml) and Cobas Amplicor HCV Monitor Assay (Roche Diagnostics) according to the manufacturer’s instructions.

### 3. Measurement of Cytokine Production

Peripheral blood samples were collected before and 1, 3 and 6 months after PEG-IFN/RBV treatment. Baseline was defined as the time of antiviral treatment introduction. PBMC were isolated on Ficoll-Hypaque gradient (Pharmacia, Sweden). One million cells were stimulated with 1 µg/ml LPS *E. Coli* 0127:B8, a natural ligand for TLR-4 or 25 ng/ml of PMA and 1 µg/ml of Ionomycin (all from Sigma-Aldrich) in RPMI medium containing 10% FCS (Gibco, Life Technologies) for 24 hours at 37°C. After stimulation, supernatants were separated by centrifugation and immediately assayed. The Human Th1/Th2 Cytokine CBA Kit (BD Biosciences) was used to quantitatively measure IL-2, IL-4, IL-6, IL-10, TNF- α and IFN-γ production according to the manufacturer’s instructions. Each specimen was measured in duplicates by FACSCalibur cytometer; data were analyzed by FCAP Array software (Soft Flow Hungary Kft.).

To verify that mainly monocytes were activated by TLR-4 ligand and they were responsible for LPS induced proinflammatory cytokine production, the effect of TLR-4 ligand (LPS) was analyzed using highly purified (>95%) T, NK cell and monocyte populations. CD14+ monocyte, CD3+ T cell and CD56+ NK cell populations were obtained from healthy control PBMC by magnetic bead separation using one step positive selection with anti-CD14, anti-CD3 and anti-CD56 coated magnetic microbeads (MACS Miltenyi Biotec, Germany). Our results showed that CD14+ monocytes were responsible for the major proportion of TLR-4 activation induced cytokine production. LPS treatment of CD3+ T and CD56+ NK cells did not result in measurable cytokine response. PMA/Ionomycin treatment activated both CD3+ T and CD56+ NK cells, but had no effect on the cytokine production of CD14+ monocytes (data not shown).

Surface staining was performed using monoclonal anti-CD4, anti-CD8, anti-CD25, anti-HLA-DR, anti-CD45RA, anti-CD45RO, anti-CD19, anti-CD56 and anti-CD14 antibodies (Pharmingen BD), then cells were analyzed by BD Calibur Flow Cytometer.

### 4. Statistical Analysis

Statistical analysis was performed using non-parametric Mann-Whitney U-test with statistical software SPSS version 11.0 package (SPSS, Inc. Chicago, IL). Results are expressed as mean value ± standard error of the mean. Correlation between variables was assessed by calculating Spearman rank correlation coefficient. Differences were accepted as significant at a level of P<0,05.

## Results

### 1. Baseline Characteristics and Phenotypes of Peripheral Blood Mononuclear Cells in Rapid Virological Responders, Complete Early- and Null-responder Patients

Pretreatment HCV RNA levels were significantly lower in rapid virological responders compared to complete early responders and null-responders. Age, BMI and liver histology data were similar in the investigated groups (Table 1.). The absolute monocyte count was slightly increased in rapid responders (p<0,05) compared to complete early- and null-responder patients. No difference was observed between peripheral blood lymphocyte phenotypes (percentage of T helper cells, cytotoxic T cells, activated T cells, memory and naïve T cells, B cells, NK cells) in the study groups (Table 2).

**Table 2:** Peripheral blood mononuclear cell phenotype characteristics in different study groups before antiviral treatment.

	RVR	cEVR	NR
**Cell count/ml**			
Neutrophilgranulocytes	2894±1073	3129±750	3159±1704
Monocytes	563±180*	483±75	414±236
Lymphocytes	1863±698	2018±399	1730±497
**Percentage of PBL**			
CD3+	77±5,3	74,5±6,7	72,3±7,2
CD4+	45,8±4,9	45,3±6,2	46,4±5,4
CD8+	28,1±5,9	25,7±7,2	24±8,1
CD3/CD25+	17,5±9,5	22,5±7,9	21,4±10,4
HLA-DR/CD3+	5,86±4,2	6,29±5,2	7,75±3,8
HLA-DR/CD8+	3,28±2,2	3,27±2,2	4,6±3,1
CD3/CD45RA+	31,63±10,9	34,6±11,7	30,1±12
CD3/CD45RO+	45,61±10,9	43,1±6,5	45,5±9,4
CD19+	8,9±3,6	13,1±6	11,8±5,2
CD56+	8,17±3	10±4,1	11±5,4

The distribution of peripheral blood lymphocyte subsets was similar between study groups. Absolute monocyte count was significantly increased in rapid virological responders compared to early and non-responder patients. Results are expressed as mean±SE.

* p<0,05.

### 2. Baseline Proinflammatory Cytokine Production of TLR-4 activated Peripheral Blood Monocytes is Increased in Rapid Virological Responders and also in Patients who Achieved SVR

**Figure 1:**
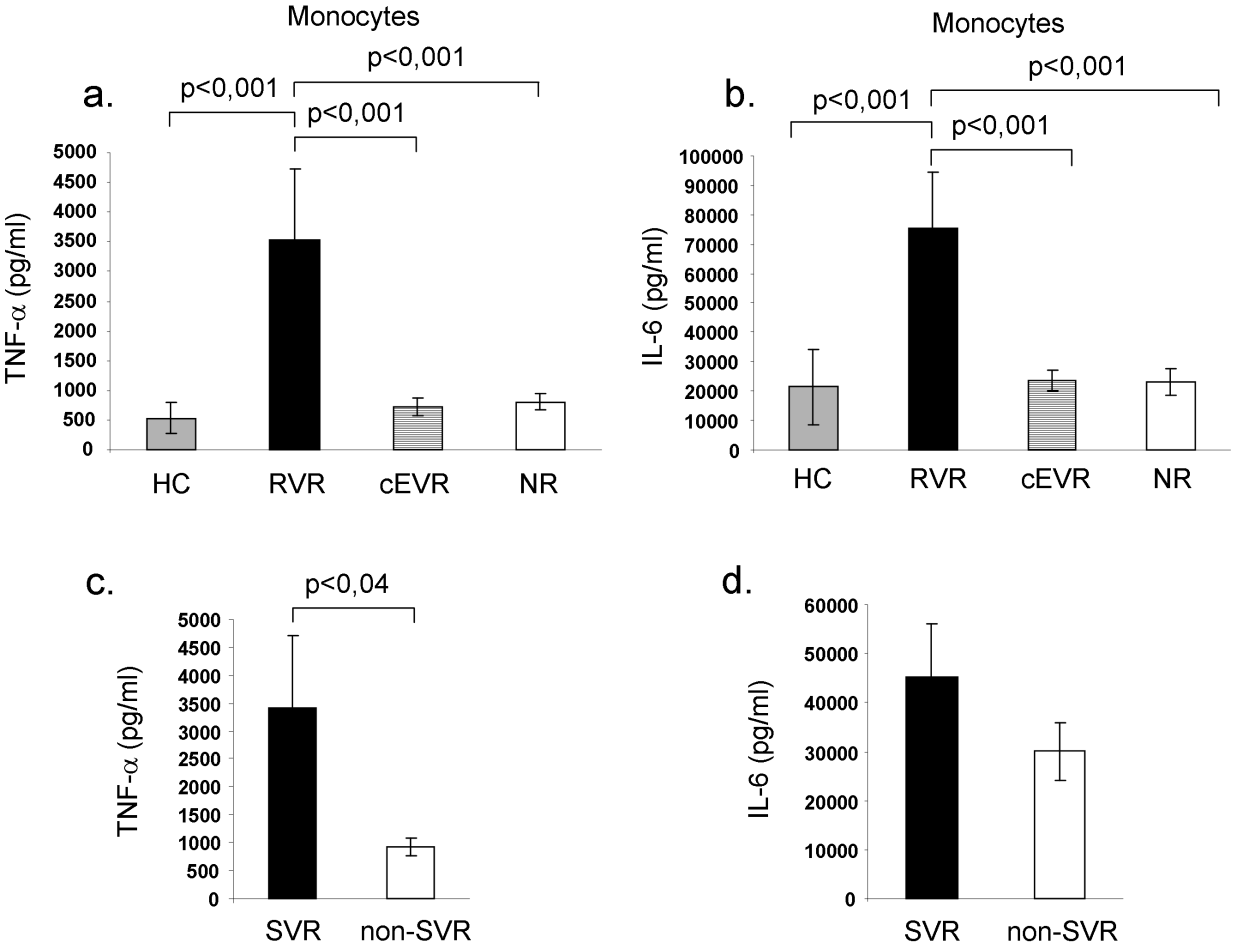
Pretreatment proinflammatory cytokine production by Toll-like receptor 4 stimulated monocytes. Prior to antiviral treatment, TLR-4 agonist induced TNF-α and IL-6 production by peripheral blood monocytes was significantly higher in later rapid virological responder CHC patients (RVR n = 14) compared to complete early virological responders (cEVR n = 19), null-responders (NR n = 17) or healthy controls (HC n = 20) (Fig. 1a,b). Baseline TLR-4 agonist induced proinflammatory cytokine production was similar in cEVR and NR groups. Sustained virological responders (SVR) had significantly higher baseline TNF-α production compared to patients without SVR (non-SVR).

Prior to initiation of antiviral therapy, TLR-4 activation of peripheral blood monocytes induced significantly higher TNF-α and IL-6 production in patients who later achieved rapid virological response compared to complete early responders, null-responders as well as healthy controls. SVR was also associated with significantly higher baseline TNF-α production compared to non-SVR group as well as healthy controls (p<0,02) (Fig. 1.).

LPS (TLR-4 ligand) induced IL-10 production did not differ significantly between the study groups, but a tendency for higher cytokine production by monocytes of RVR patient was observed. (RVR: 1580±300 pg/ml, cEVR: 1030±250 pg/ml, NR: 900±250 pg/ml, healthy control:1712±250 pg/ml, SVR: 1199±461 pg/ml, non-SVR: 1212±242 pg/ml). IL-2, IL-4 and IFN- γ production by the LPS stimulated monocytes were under the detection limit of CBA Assay.

These data suggest that monocytes of rapid virological responders have strong potential to produce proinflammatory cytokines (TNF-α, IL-6) after TLR-4 activation compared to patients without rapid virological response. Patients who later achieved SVR had also higher baseline TNF-α production compared to non-SVR group (Fig. 1c.).

### 3. Proinflammatory Cytokine Secretion by TLR-4 Activated Monocytes is Enhanced by PEG-IFN/RBV Treatment in Complete Early Virological Responders

**Figure 2:**
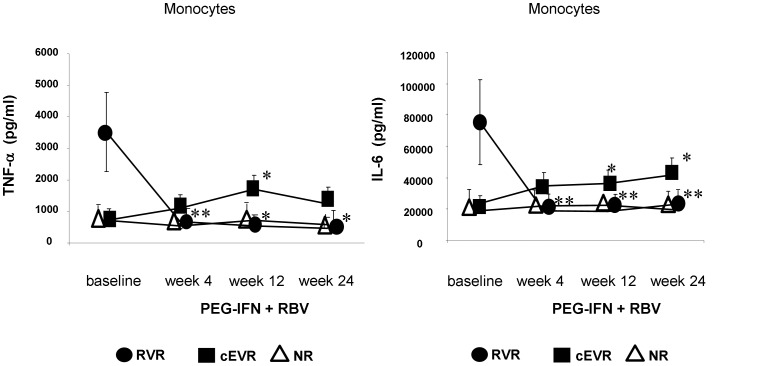
The effect of PEG-IFN plus ribavirin treatment on TNF-α and IL-6 production by TLR-4 stimulated monocytes. After 12 weeks of PEG-IFN plus RBV treatment, the proinflammatory cytokine production of TLR-4 stimulated monocytes was significantly increased in complete early virological responders compared to null-responder patients. Furthermore, proinflammatory cytokine levels showed no changes and remained low in null-responders throughout antiviral therapy. In contrast to cEVR in RVR patients, proinflammatory cytokine production by monocytes was significantly decreased after 4 weeks of treatment (*p<0,05; **p<0,01 compared to baseline values).

While 12 weeks of PEG-IFN/RBV treatment significantly increased TNF-α and IL-6 production of TLR-4 activated monocytes in cEVR patients, proinflammatory cytokine production remained at low levels in null-responders. Interestingly, after 4 weeks of treatment - when viral clearance was achieved - a dramatic decrease of TLR-4 agonist induced proinflammatory cytokine production was detected in RVR patients. In RVR patients high baseline level of IL-6 and TNF-α decreased to a comparable level found in null-responders, suggesting that elevated inducible baseline TNF-α, IL-6 production of monocytes may depend on the presence of the virus (Fig. 2.). During antiviral treatment, IL-10 production by monocytes showed no remarkable changes in any of the study groups (data not shown).

### 4. Non-HCV Specific Activation of Peripheral Blood T Lymphocytes and NK Cells Induces Increased IFN-γ and Decreased IL-4 and IL-10 Production in Rapid Virological Responders

**Figure 3:**
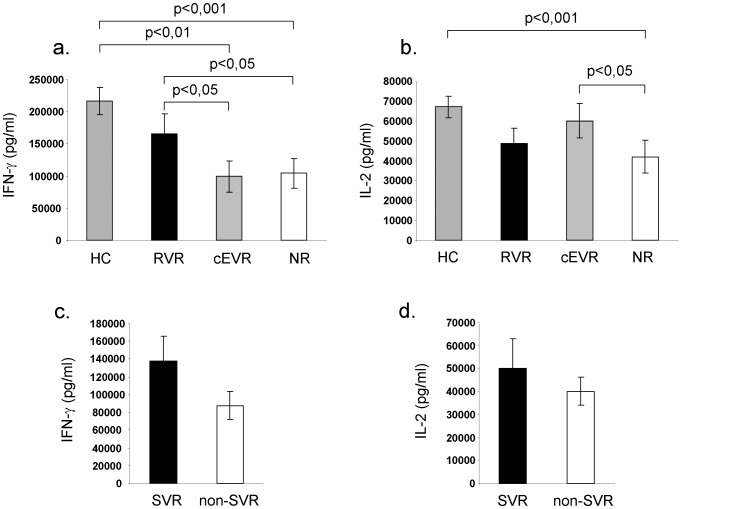
Th1 cytokine production by PMA/Ionomycin stimulated peripheral blood mononuclear cells. Rapid virological responders and healthy controls showed significantly higher IFN-γ production compared to complete early responders and null-responders. IFN-γ and IL-2 levels were significantly lower in null-responders compared to healthy controls (Fig. 3a,b.). Lymphocytes Th1 cytokine production did not differ significantly between SVR and non-SVR group (Fig. 3c,d.).

**Figure 4:**
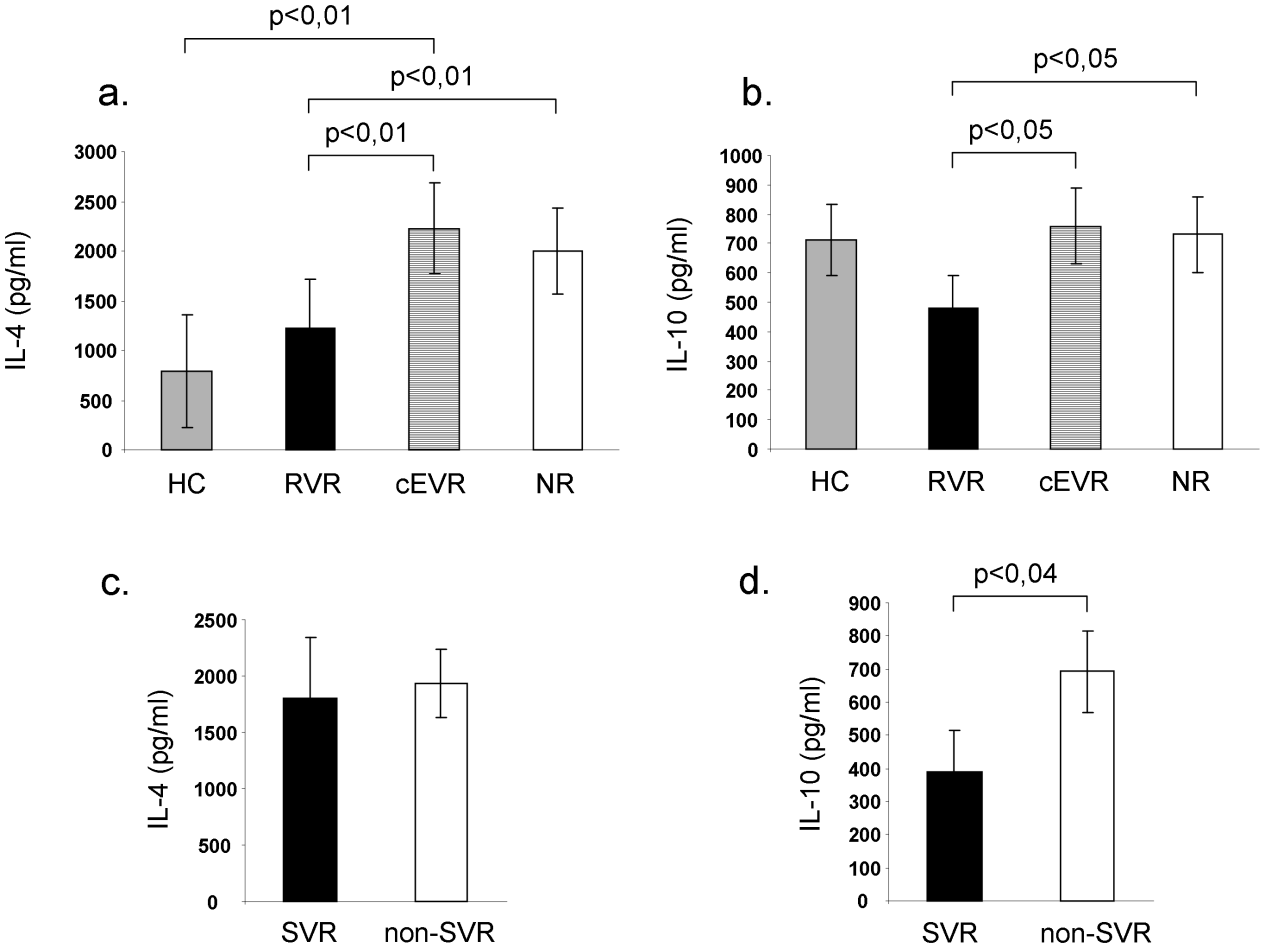
Th2 cytokine production by PMA/Ionomycin stimulated peripheral blood mononuclear cells. Prior to therapy, IL-4 and IL-10 production was significantly lower in patients who had subsequent rapid viral decline after 4 weeks of treatment compared to non-SVR group. Baseline Th2 cytokine production did not differ between complete early responders and null-responders (Fig. 4a,b). SVR patients associated with significantly lower baseline IL-10 production compared to non-SVR patients (Fig. 4d).

Rapid HCV RNA clearance was associated with increased baseline IFN-γ (Fig. 3a.) and decreased Th2 (IL-4 and IL-10) (Fig. 4a,b.) cytokine production by PMA/Ionomycin stimulated PBMC compared to non-RVR groups. SVR was also associated with significantly lower baseline IL-10 production compared to non-SVR group (Fig. 4d). We found a tendency for higher baseline IFN-γ production in patients who achieved SVR compared to non-SVR group, but the differences were statistically not significant (Fig. 3c.).

While baseline IL-2 production by the PMA/Ionomycin activated PBMC was significantly higher in cEVR compared to NR patients (Fig. 3b.), no differences were found in IL-6 and TNF-α production between study groups (IL-6: RVR:2860±840 pg/ml, cEVR:4090±1310 pg/ml, NR:3880±960 pg/ml, healthy control:4057±680 pg/ml, SVR:3958±1069 pg/ml, non-SVR: 4445±922 pg/ml and TNF-α: RVR:8060±1830 pg/ml, cEVR:10910±1950 pg/ml, NR:11420±2030 pg/ml, healthy control:7715±1000 pg/ml, SVR:10631±2084 pg/ml, non-SVR:8967±1272 pg/ml). Peripheral blood mononuclear cells of RVR patients produced significantly lower baseline levels of IL-4 and IL-10 compared to non-RVR groups (Fig. 4a,b.). Prior to P/R treatment Th2 cytokine production of the lymphocytes did not differ between cEVR and NR groups. While IFN-γ, IL-2, IL-4 and IL-10 production by lymphocytes did not differ between RVR patients and healthy controls, Th1 type cytokine production (such as IFN-γ, IL-2) by null-responders were significantly lower than in healthy controls (Fig. 3a,b.).

### 5. The Effect of PEG-IFN/RBV Treatment on Th1/Th2 Cytokine Production of PMA/Ionomycin Activated Mononuclear Cells

**Figure 5:**
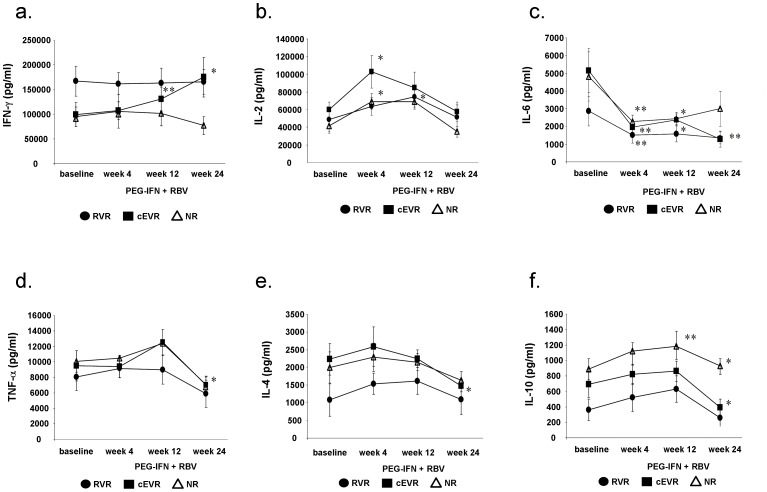
The effect of PEG-IFN plus ribavirin treatment on Th1/Th2 cytokine production by PMA/Ionomycin stimulated PBMC. a. IFN-γ production was significantly increased in complete early virological responders during antiviral treatment compared to pretreatment levels and also to null-responders. b. After 4 and 12 weeks of antiviral treatment, a transient increase in IL-2 production was observed in all study groups. c. PEG-IFN plus RBV resulted in decreased IL-6 production in both rapid and complete early virological responders, had a transient effect in null-responders. d. After 24 weeks of treatment, significantly decreased TNF-α production was found in complete early virological responders. e. f. While PEG-IFN/RBV treatment significantly decreased IL-4 and IL-10 levels in complete early virological responders, null-responders showed significantly increased IL-10 production at week 12 or 24. (*p<0,05; **p<0,01).

While PEG-IFN/RBV treatment significantly increased IFN-γ production by lymphocytes in patients with cEVR, in null-responders low IFN-γ production was maintained. At the sixth month of antiviral treatment not only RVR, but also cEVR patients had significantly higher level of IFN-γ compared to null-responders (Fig. 5a.). After 4 weeks of treatment notable increase in IL-2 production of PMA/Ionomycin activated PBMC was also observed, especially in the cEVR group (Fig. 5b.). Antiviral treatment decreased IL-6 production by lymphocytes in both RVR and cEVR group and had only transient effect in null-responders (Fig. 5c.). After 6 months of antiviral treatment, TNF-α production decreased in all study groups (Fig. 5d.). Furthermore, after 24 weeks of PEG-IFN/RBV treatment a significant decrease of Th2 cytokine production (IL-4, IL-10) by PBMC was observed in cEVR patients compared to NR patients (Fig. 5e,f.). At this timepoint IL-4 and IL-10 production of cEVR were equally low as of RVR patients. After 12 and 24 weeks of PEG-IFN/RBV treatment, IL-10 production by PBMC was significantly higher in null-responders compared to their baseline levels (Fig. 5f.).

### 6. Correlation between Cytokine Production and Patients’ Baseline Characteristics

No correlation was found between pretreatment patients’ characteristics (HCV RNA levels, gender, BMI, HAI, fibrosis, ALT) and Th1/Th2 cytokine production. A positive correlation was found between TLR-4 ligand induced TNF-α and IL-6 production by monocytes (r = 0,78 p<0,01) and also between IL-4 and IL-10 secretion by PMA/Ionomycin stimulated PBMC (r = 0,68 p<0,01).

## Discussion

Rapid viral decline over four weeks of P/R treatment has been suggested as the strongest on-treatment predictor of SVR [23], [24]. The immunological background of RVR has not been completely clarified. The present study shows evidence that non-antigen specific activation of both innate and adaptive immune cells result in increased baseline proinflammatory cytokine production in future rapid virological responders compared to non-RVR chronic hepatitis C patients. Elevated TNF- α and IL-6 production by TLR-4 activated monocytes, increased IFN-γ and diminished IL-4 and IL-10 production by PMA/Ionomycin stimulated T lymphocytes and NK cells at baseline were pre-treatment indicators of RVR. We also found increased baseline TNF-α production with low IL-10 levels in patients who later achieved SVR compared to non-SVR group.

Several data suggest that preactivation of the endogenous IFN system is strongly linked to the later non-response to treatment, while low initial ISG expression at baseline and increased HCV specific CD4+ and CD8+ T-cell reactivity are associated with RVR [12], [25].

Recent data suggest that IL28B polymorphism, low HCV RNA and low IP-10 levels independently predict RVR [26].

Several studies using different cell activation methods demonstrated conflicting results regarding the altered cytokine production in chronic HCV infection. Activation of TNF-α system or poor HCV specific Th1 cytokine responses with Th2 cytokine dominance (e.g. up-regulation of IL-10 by monocytes) has been described [22], [27]. The positive impact of the ribavirin-induced Th2/Th1 cytokine shift towards Th1 cytokine production has also been described in CHC patients [28], [29].

While high baseline levels of IL-10 and low levels of IL-12p40 were correlated with IL28B gene polymorphism and associated with NVR; high levels of interleukin IL-12 and IL-18 were associated with SVR [18], [19].

A growing body of evidence suggests changes in Toll-like receptor signaling pathways (e.g. blockade of TLR-3, RIG-I signaling) and expression levels of TLR mRNAs in CHC patients with or without responsiveness to antiviral therapy [30]–[36]. Up-regulated gene expression of TLR-3, TLR-4, TLR-7 and enhanced expression of TLR signaling molecules by IFN-α have also been described [30], [37]. While HCV genotype 1 infected non-responder patients with cirrhosis showed increased LPS receptor (sCD14) levels, HIV/HCV co-infected patients sCD14 (LPS receptor) levels correlated also with the severity of liver disease and predicted unfavorable response to P/R which suggests that in non-responders an overall higher innate immune activation with higher endogenous IFN production might be present, which might render the immune cells less sensitive to exogenous IFN [38]. Li et al. demonstrated that activation of chemokine and inflammatory cytokine response in HCV infected hepatocytes depends on TLR-3 sensing of HCV double-stranded RNA intermediates. [39].

We now report that prior to treatment, TLR-4 activation induced proinflammatory cytokine production by monocytes is associated with RVR and consequent SVR, and assume that signal mechanisms through TLRs may play a crucial role in the responsiveness to P/R therapy. Since TLR-4 shares common signal transduction pathways with other TLRs (e.g. TLR-3, TLR-7, TLR-9) [22], the ineffective activation of other TLRs by dsRNA or damage associated molecular patterns resulting in impaired induction of IRF-3, IFN-β in non-responders with altered proinflammatory cytokine production may be hypothesized. A model of innate immune escape by HCV involving limited initial induction and stringent subsequent control of the IRF-3 response supports this hypothesis [40]. Hepatic gene expression profiling has also demonstrated an upregulated and largely ineffective IFN response in non-responders, and raised the possibility of active downstream inhibitors that render an ineffective endogenous and exogenous IFN response [41]. Data suggest that in CHC patients TLR-3 and TLR-4 innate sensing functions of circulating human myeloid dendritic cells are affected and assumed that HCV generates mature dendritic cells that stimulate Th2 cells. This impairment of pathogen recognition receptor-induced proinflammatory cytokine production is intracellular HCV RNA density dependent [42]. In line with this, we also found that in non-responders high baseline HCV RNA levels were associated with defective TLR-4 activation induced proinflammatory cytokine production and a Th2 cytokine dominance.

The potential weakness of the study is that we did not analyze cytokine production after activation other TLR-s such as TLR7 or TLR9 which are also important in antiviral response. Although measurement of baseline TNF-α production by monocytes may be useful in predicting RVR or more important SVR, the CBA cytokine assay technology applied can not be expected to be used in routine clinical practice, since its protocol is based on live cell stimulation and flow cytometry.

Since cytokines produced by innate immune cells (such as monocytes, NK-, dendritic cells) are thought to be key regulators of adaptive Th1/Th2 responses, it is hypothesized that in patients with the lack of virological response, monocytes do not initiate effective proinflammatory antiviral immune response and contribute to viral replication. Our observation supports this assumption, since we showed an increased proinflammatory cytokine production by monocytes in cEVR patients after viral clearance (at week 12) associated with increased IFN-γ and decreased Th2 cytokine levels by lymphocytes. In contrast to cEVR patients, low proinflammatory cytokine production by monocytes and elevated IL-4 and IL-10 production by T lymphocytes and NK cells were maintained in null-responders throughout the treatment.

Since liver infiltrating immune cells can contribute to hepatic ISG or immunological gene expression [43], the observed increased IL-10 production by null-responders and patients who later did not achieve SVR may result in the dysfunction of intrahepatic virus-specific T cells, which facilitate viral persistence described in other viral infections [44]–[46].

Interestingly, the clearance of the virus abolished the high proinflammatory cytokine production capacity of LPS stimulated monocytes in rapid responders suggesting that the presence of the virus is essential for monocytes to alter sensitivity to TLR-4 stimulation. In contrast to monocytes, increased baseline IFN-γ production by T lymphocytes and NK cells was maintained during antiviral treatment in rapid responders. An enhanced IFN- γ production was observed after viraemia clearance in complete early responders compared to null-responders. P/R treatment decreased IL-6 production by lymphocytes in RVR and cEVR patients and had only transient effect in null-responders. Since correlation between serum IL-6 levels and liver injury (HAI) has been described [47], [48], antiviral treatment induced decreased IL-6 production in virological responders may indicate a decline in hepatic inflammation and support the assumption that achievement of SVR after IFN-α therapy is associated with an improved outcome in liver-related mortality [49], [50].

In conclusion, our study provides evidence that baseline Th1/Th2 cytokine production by both innate and adaptive immune cells differs in rapid responders compared to complete early virological responders and null-responders. RVR was associated with increased baseline TNF-α, IL-6 production by TLR-4 activated monocytes, increased IFN-γ and decreased IL-4, IL-10 production by T lymphocytes and NK cells compared to cEVR and NR. Patients who achieved SVR also had significantly higher TNF-α and lower IL-10 production compared to non-SVR patients. TLR-4 sensing function and proinflammatory cytokine production were impaired in null-responders supporting that modulation of TLR activity and cytokine production could have beneficial effects in these patients. The differences observed in TLR-4 ligand induced activation of monocytes between RVR and non-RVR patients suggest that TLR signaling and consequent induction of endogenous IFNs and IFN-stimulated gene products are important to determine antiviral treatment response. Unfortunately, previous oral TLR-7 agonist therapy of CHC patients was associated with serious adverse events, raising concerns about the therapeutic use of this class of compounds for HCV infection [51].

Our data suggest that investigating the differences in TLR signaling as well as factors determining antiviral cytokine production in HCV infection would help to develop new immunotherapeutic approaches potentiating the effectiveness of currently used antiviral therapy, especially in null-responder patients.
